# Baxter Physioneal, Extraneal, Nutrineal (PEN) and Dianeal solution bags can be accidentally connected to Fresenius peritoneal dialysis catheter extensions in a non-sterile manner

**DOI:** 10.1093/ckj/sfae067

**Published:** 2024-03-14

**Authors:** Annemarie Albert, Stefan Richter, Rainer Peter Woitas, Ulrich Paul Hinkel, Philipp Stieger, Rüdiger C Braun-Dullaeus, Christian Albert

**Affiliations:** Diaverum Renal Services, Potsdam, Germany; Department of Nephrology, Central Clinic Bad Berka, Bad Berka, Germany; Diaverum Renal Services, Potsdam, Germany; Diaverum Renal Services, Potsdam, Germany; Department of Nephrology, Central Clinic Bad Berka, Bad Berka, Germany; University Clinic for Cardiology and Angiology, Otto-von-Guericke University Magdeburg, Magdeburg, Germany; University Clinic for Cardiology and Angiology, Otto-von-Guericke University Magdeburg, Magdeburg, Germany; Diaverum Renal Services, Potsdam, Germany; Department of Nephrology, Central Clinic Bad Berka, Bad Berka, Germany; University Clinic for Cardiology and Angiology, Otto-von-Guericke University Magdeburg, Magdeburg, Germany

To the Editor,

Peritoneal dialysis (PD) is an important and increasingly provided renal replacement therapy for patients with end-stage renal disease, enabling the provision of a home-based therapy. In the Americas and Europe, a small number of systems are available for the provision of PD, only, and in general the companies’ proprietary connection systems are not compatible with each other [1].

Persons doing their PD independently at home are occasionally faced with medical or procedural problems [2]. One of our patients doing continuous ambulatory PD (CAPD) on the Baxter (BX) system presented to our centre 1 day after hospital discharge. At home he followed his prescription plan using Physioneal (BX). After his bag exchange, he found that the Minicap would not fit his transfer set and he reported puddles of water on the floor. At inspection we recognized that his transfer set connection was still based on the Fresenius medical care (FMC) stay-safe system intended for use with the FMC DISC [3], which is luer-lock- and PIN-based. We determined that at hospital admission, the patient’s transfer set was changed from BX to FMC, however the hospital PD provider forgot to change the patient’s transfer connection back to BX before discharge (Fig. 1A).

**Figure 1:**
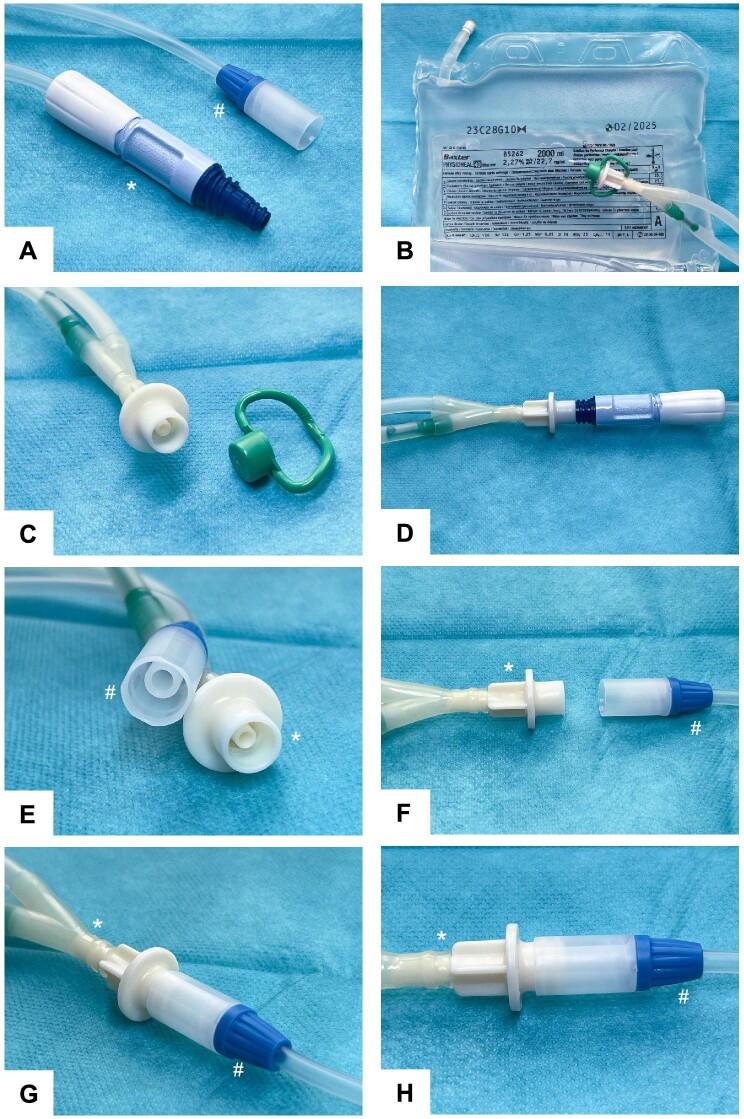
(**A**) *BX PD transfer set and #FMC stay-safe catheter extension or transfer set. (**B**) Exemplary BX solution for CAPD from the PEN series which all share the same double bag lines and Y-set connector. (**C**) PEN solution to BX transfer set connector with double bag lines. (**D**) Intended BX PEN to BX transfer set connection. (For illustrational purpose without iodized disinfection cuff.) (**E** and **F**) Close-up view of FMC stay-safe catheter extension (#, transfer set) and BX PEN solution connector (*). (**G** and **H**) BX PEN solution (*) connected to an FMC stay-safe catheter extension (#). The parts of this connection are easy to plug into each other. It is not luer-locked as both systems are not compatible, and therefore it is unsealed and prone to leakage. The attachment is potentially unsterile as the outer part of the PEN bag connector attaches into the FMC connector and is exposed to the dialysate solution if used. Therefore, such connection of BX PEN solutions to FMC transfer sets is not safe for use. All materials shown in Fig. 1 have no reference to an individual patient and were taken from routine clinical consumables for illustrational purpose.

The patient cut the bag’s Y-set and clamped the transfer set for safety (Supplementary data, Fig. S1). To our surprise, a plug-in connection between the FMC stay-safe transfer set and BX Physioneal, Extraneal, Nutrineal (PEN [4]) and Dianeal CAPD solutions was possible without tampering (Fig. 1E–H). In our patient, the FMC transfer set was exchanged for a BX set under sterile conditions. We collected dialysate cultures from the initial drain and administered intraperitoneal vancomycin and ceftazidime. The case was observed thoroughly until cultures reported negative. No side effects occurred.

We would like to draw attention to this issue and stress that BX PEN solution connection to FMC transfer sets is unintended, not interlocked, unsealed and prone to leakage and unsterile, and therefore not safe for use.

All Baxter PEN solutions and Dianeal for CAPD use a standardized Y‐set and double bag exchange system [5], and connect to BX transfer sets via a male-to-female luer-lock connection. Using the non-touch technique, the outer part of the PEN solution connection will not come in contact with the luer-lock system of the transfer set (Fig. 1D). FMC stay-safe is based on luer-lock connections incompatible with that of BX. However, the same methodology applies to the FMC stay-safe transfer set, where the inner part of the stay-safe connector is never brought into contact with a potentially unsterile part of the DISC system, to prevent contamination. After each bag exchange the FMC transfer connector is secured with a PIN, inserted at the last step of the DISC use procedure and a luer-locked stay-safe disinfection cap.

When unintentionally connecting BX PEN solution bags to an FMC transfer set, the outer part of the bag connector will interact with the inner part of the FMC stay-safe connection, with the potential risk of contamination. Additionally, the dialysate solution will then flow through this unsecured connection, with an additional risk for leakage and disconnection as no appropriate luer-lock safety mechanism is applied.

We suggest that specifically older persons doing PD and those who do not perform PD by themselves (assisted PD) may be at increased risk of misconnecting the two companies’ PD systems. To ensure patients’ safety when performing PD and to minimize the potential risk of contamination and peritonitis, we recommend that all patients be switched back to their previous company's delivery system by the hospitals’ PD provider before hospital discharge and that appropriate consultation be held with the responsible outpatient dialysis centre.

## Supplementary Material

sfae067_Supplemental_File
