# Supplementary material for: Baxter Physioneal, Extraneal, Nutrineal (PEN) and Dianeal solution bags can be accidentally connected to Fresenius peritoneal dialysis catheter extensions in a non-sterile manner
Source: Clin Kidney J. 2024 Mar 14;17(4):sfae067. doi: 10.1093/ckj/sfae067 (PMC11015149; doi:10.1093/ckj/sfae067)
Supplement: sfae067_Supplemental_File [file sfae067_supplemental_file.docx]

**Supplementary Material**

**Baxter Physioneal, Extraneal and Nutrineal (PEN) solution bags can be accidentally connected to Fresenius peritoneal dialysis catheter extensions in a non-sterile manner.**

Annemarie Albert^1,2^, Stefan Richter^1^, Rainer Peter Woitas^1^, Ulrich Paul Hinkel^2^, Philipp Stieger^3^, Rüdiger C. Braun-Dullaeus^3^, Christian Albert^1,2,3^*

^1^ Diaverum Renal Services, Potsdam, Germany

^2^ Department of Nephrology, Central Clinic Bad Berka, Bad Berka, Germany

^3^ University Clinic for Cardiology and Angiology, Otto-von-Guericke University Magdeburg, Germany

* **Address for Correspondence:**

Christian Albert

University Clinic for Cardiology and Angiology, Otto-von-Guericke University Magdeburg, Leipziger Str. 44, 39120 Magdeburg, Germany


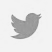
E-mail: Christian.Albert@med.ovgu.de

@TheKidneyChris

**Supplementary Figures**

**
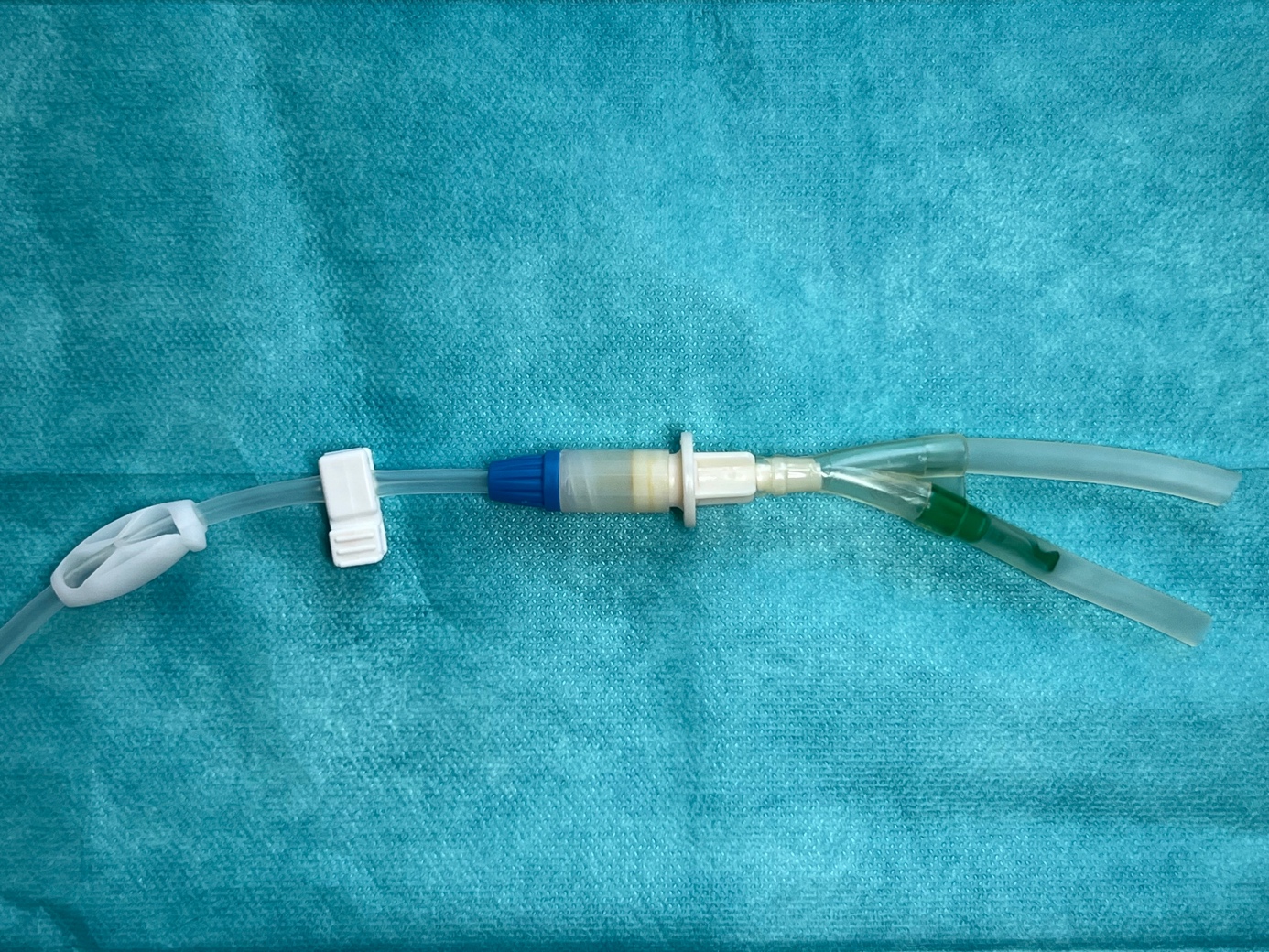
**

%

$

§

#

*

**Supplementary Figure 1:** Reproduction of the patients transfer system at presentation at our outpatient clinic. The transfer set clamp ($) is closed. An additional clamp (§) is in place. FMC Transfer set connector (#), BX PEN bag connector (*) with cut Y-lines (%).

**Additional figures illustrating the intended connection procedures of BX and FMC material.**

**
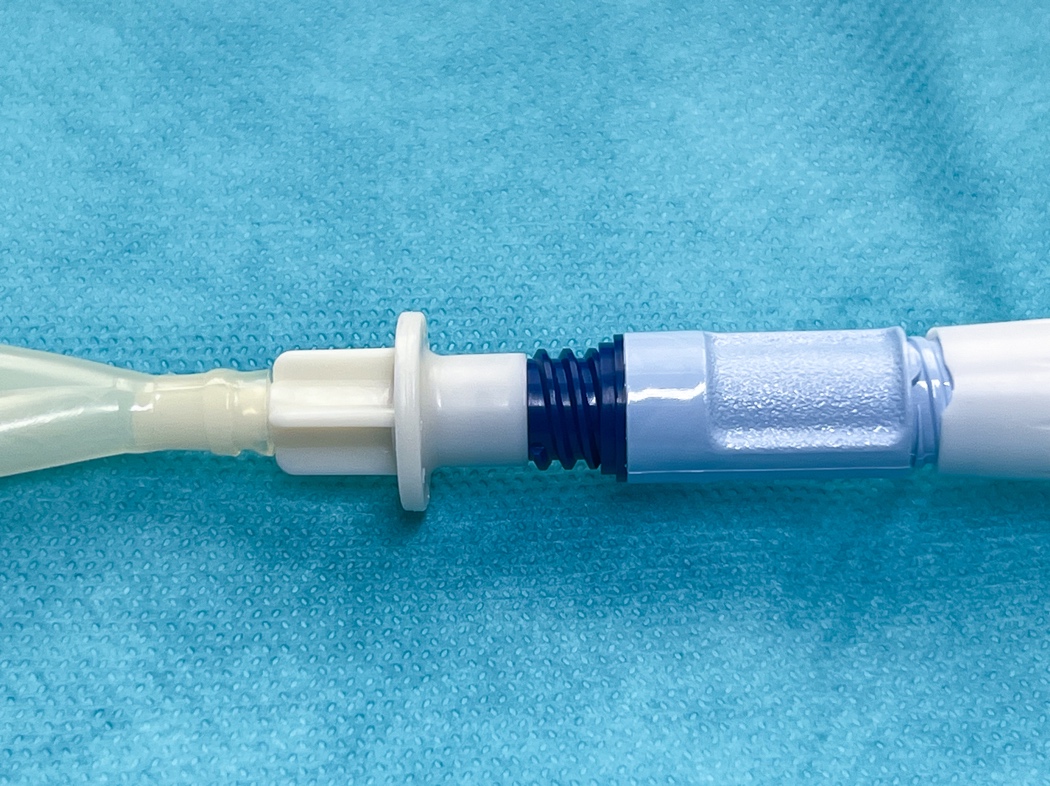
**

**Supplementary Figure 2:** Close up of intended BX PEN/Dianeal to BX transfer set connection. For illustrational purpose shown without iodized disinfection cuff in place.


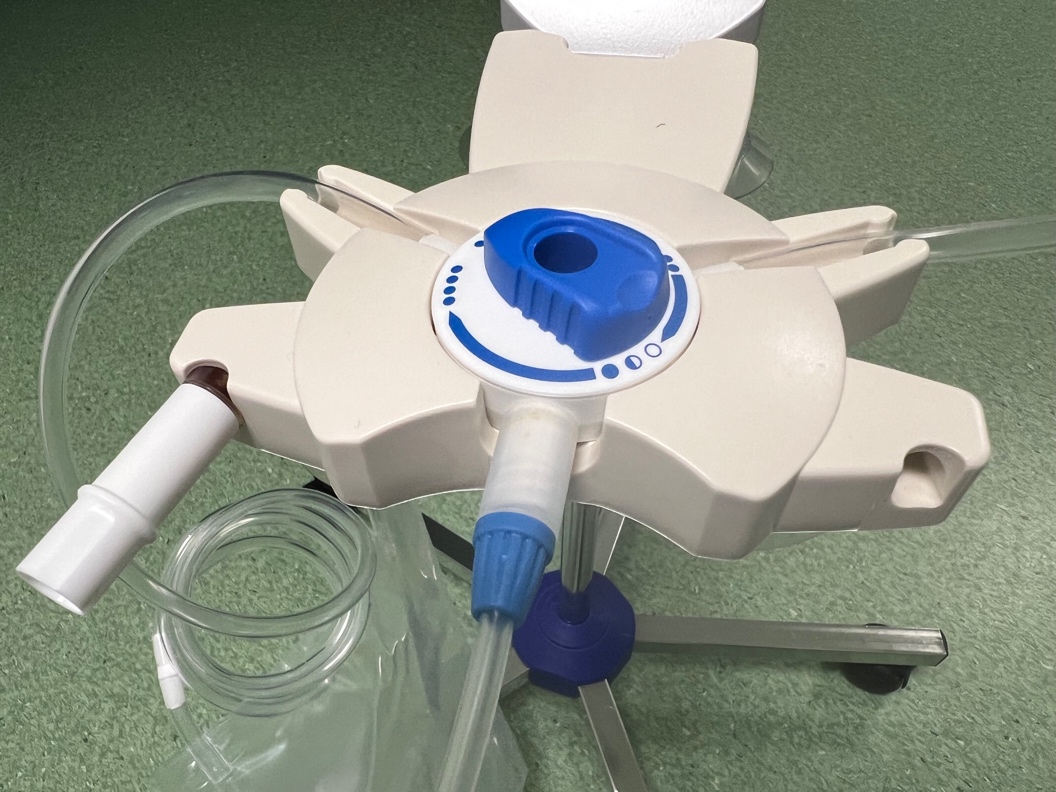


**Supplementary Figure 3:** Intended use of FMC stay•safe patient transfer connection to the FMC DISC device for bag exchange. After a complete clockwise turn of the DISC indicator a PIN is loaded into the transfer set connector which then locks into the iodized cap (on the left side of the DISC) sealing the catheter extension after disconnection.

**Reference to relevant materials**

**FMC (Fresenius Medical Care AG & Co. KGaA, 61346 Bad Homburg, Germany)**

Stay•safe Catheter Extension Luer-Lock 40cm REF 2843251 Art. 284 325 1

Stay•safe Desinfection Cap REF 2845091 Art. 284 509 1

**BX (Baxter Healthcare SA, 8010 Zürich, Switzerland)**

MiniCap Extended Life PD Transfer Set with Twist Clamp REF R5C4482

MiniCap REF SPC4466

Baxter Physioneal 40 Glucose 2.27% / 22.7mg/mL, 2L bag HPB5262

All BX PEN and Dianeal bags in Europe and the US share the same connector.

**Adapting PD catheter connections to alternate PD delivery systems available in the North American market**

Specifically, in North America small adapter sets for BX PEN to FMC stay-safe PD and FMC stay-safe to BX PEN are available from FMC North America (FMCNA) which is particularly useful for hospitalized patients, since there is no need to perform a transfer system exchange. We contacted FMC (Bad Homburg, Germany) who confirmed, that these adapters are approved for the use in the North American market, only. For the European market however, an FMC adapter for the connection of Extraneal (BX) is available for a single-bag exchange use with the CAPD DISC. Additionally, an adapter intended for single-use of Extraneal as a last bag option with the sleep-safe harmony cycler (FMC) is available. However, both are not licensed for multiple use.

**BX to FMCNA / FMCNA to BX adapter sets**

*Baxter Patient transfer set to FMCNA stay•safe PD-disc system:*

Connect FMCNA stay•safe luer-lock 6-inch extension set (FMCNA Product No. 050-95013) to transfer set.

*FMCNA stay•safe Patient transfer set to Baxter PEN system:*

Connect FMCNA luer-lock adapter 4-inch (FMCNA Product No. 050-95003) to FMCNA stay•safe extension sets then connect Baxter transfer set to the FMCNA luer-lock adapter.

**FMC to BX single use adapter available in Europe intended for use with Icodextrin (Extraneal, BX)**

Stay•safe Luer-Lock Set (CAPD) REF 259991 Art. 259 991 1

Sleep•safe Luer-Lock Adapter (APD) Art. 501 697 1

*Abbreviations*: FMCNA, Fresenius Medical Care North America; PEN, Physioneal, Extraneal, Nutrineal solutions; PD, Peritoneal Dialysis; BX, Baxter

Art. Numbers refer to the German FMC Products and Services catalogue valid from 2020.

All materials shown in the Supplementary Figures have no direct reference to an individual patient and were taken from routine clinical consumables for illustrational purpose.
